# Epidemiological cohort study of systemic antifungal therapy for suspected or confirmed invasive candidiasis in the ICU: the Amarcand2 study

**DOI:** 10.1186/cc14191

**Published:** 2015-03-16

**Authors:** J Constantin, JF Timsit, JP Gangneux, JP Mira, P Montravers, H Dupont, P Perrigault, O Lortholary, E Azoulay, O Leroy

**Affiliations:** 1 CHU Estaing, Clermont-Ferrand, France; 2 Paris Diderot University/Bichat Hospital, Paris, France; 3 Rennes University Hospital, Rennes, France; 4 Cochin University Hospital, Paris, France; 5 Amiens University Hospital, Amiens, France; 6 Montpellier University Hospital, Montpellier, France; 7 Necker University Hospital, Paris, France; 8 Saint-Louis University Hospital, Paris, France; 9 Tourcoing University Hospital, Tourcoing, France

## Introduction

Prescription of antifungal treatments (AFT) in ICUs in case of suspected or confirmed invasive candidiasis (SIC or CIC) has been challenged by different guidelines. The study aimed to describe the epidemiology of the invasive candidiasis (IC), analyze the criteria for the AFT initiation, the AFT type, and its changes during patient follow-up.

## Methods

A prospective observational multicenter cohort study. Consecutive adult patients with SIC or CIC and treated with systemic AFT were included between October 2012 and September 2013 in 104 French ICUs.

## Results

In total, 870 patients were included and 835 evaluable, the IC was confirmed at study inclusion for 291 and suspected for 544 patients. Eventually, the IC was confirmed for 403/835 patients (peritonitis: 177; candidaemia: 141; deep candidiasis: 61; mixed infection sites: 24). *Candida albicans *was the main pathogen (67%), then *C. glabrata *(16%). At inclusion, CIC were treated with caspofungin (Cas): 55%, and fluconazole (Flu): 34%, whereas these antifungals were administered to 46% and 45% of SIC, respectively. Patients with SIC were more severe than those with CIC. The two main criteria for initiating empirically an AFT were a central venous catheter (79%) and severe septic shock (70%). The rate of change of the initial AFT was higher in the CIC group (49%) than in the SIC group (33%, *P *< 0.0001). In the CIC group, it was mostly for changing the antifungal agent (de-escalation Cas → Flu in half of the patients) based on mycological tests results. In the SIC group, the AFT was modified almost as often for changing the drugs (including 22% de-escalation Cas → Flu) as for stopping the AFT. The 28-day mortality of candidaemia was 42% in cases of *C. glabrata*, 40% in cases of *C. albicans*, and 20% in cases of *C. parapsilosis*. Among survivors, the median duration of treatment was 17 to 21 days according to the infection site in cases of CIC, and 10 days in cases of SIC.

## Conclusion

French ICU patients are treated with antifungal agents selected according to the candidiasis severity, contrary to ESCMID guidelines which recommend initiating with echinocandins regardless of severity. As recommended, the therapy was secondarily adapted to microbiological results.

